# Association of serum 25-hydroxyvitamin D concentrations with all-cause mortality among individuals with kidney stone disease: the NHANES database prospective cohort study

**DOI:** 10.3389/fendo.2023.1207943

**Published:** 2023-10-03

**Authors:** Meng Gao, Minghui Liu, Jinbo Chen, Zewu Zhu, Hequn Chen

**Affiliations:** ^1^ Department of Urology, Xiangya Hospital, Central South University, Changsha, China; ^2^ National Clinical Research Center for Geriatric Disorders, Xiangya Hospital, Central South University, Changsha, China

**Keywords:** kidney stones, 25-hydroxyvitamin D, National Health and Nutrition Examination Survey, all-cause mortality, inflammation

## Abstract

**Background:**

The purpose of this study was to investigate the correlation between serum 25(OH)D concentrations and all-cause mortality in patients with kidney stone disease (KSD) as the effects of a deficiency in 25-hydroxyvitamin D on KSD patients are currently unclear.

**Methods:**

For our prospective cohort study, we included 2,916 participants from the National Health and Nutrition Examination Survey (NHANES) 2007-2018. The National Death Index (NDI) was utilized to identify all causes of death and cause-specific mortality until December 31, 2018. We calculated hazard ratios (HR) and 95% confidence intervals (CIs) using multivariate Cox regression models.

**Results:**

During the 18,859 person-years of follow-up, a total of 375 fatalities occurred, including 83 deaths from cardiovascular disease (CVD) and 79 deaths from cancer. At baseline, individuals with higher blood 25(OH)D concentrations had lower levels of glucose, glycohemoglobin, CRP, and insulin, as well as higher levels of HDL cholesterol (P < 0.01). In the fully adjusted model (Model 3), compared to the group with the lowest 25(OH)D concentrations, those with serum 25(OH)D concentrations ≥75 nmol/L had hazard ratios (HRs) and 95% confidence intervals (CIs) of 0.48 (0.26, 0.87) for all-cause mortality (P=0.02, P for trend = 0.02). The association between serum 25(OH)D concentrations and all-cause mortality in KSD patients was found to be significantly non-linear. A 7% decrease in the risk of death from all causes was observed for each unit-nmol/L increase in serum 25(OH)D concentrations when the concentrations were below 27.7 nmol/L (P < 0.05).

**Conclusion:**

Based on the findings, KSD patients with insufficient serum 25(OH)D concentrations were at a higher risk of all-cause mortality. Therefore, it is crucial to maintain sufficient blood 25(OH)D concentrations and prevent 25(OH)D insufficiency in order to extend the lifespan of KSD patients.

## Introduction

Kidney stone disease (KSD) is a highly prevalent and increasingly common disease worldwide, with an estimated prevalence of 1.7-14.8% (1). Recent NHANES studies have demonstrated a rise in the prevalence of KSD, particularly among women, with their prevalence increasing from 6.5% to 9.4% (2). Furthermore, KSD can affect individuals of various age groups (2, 3), and the presence of kidney stones can lead to various complications, such as renal colic, obstruction, renal function impairment, and, in severe cases, sepsis. As a result, KSD places a significant burden on the global public health system, with inpatient stays for KSD accounting for 0.5% of all hospital stays (4, 5). Currently, there are several effective treatment options available for kidney stone disease (KSD). However, one significant challenge in managing KSD is the high recurrence rate of stones after their removal (6). The cost associated with KSD exceeds $10 billion annually in the United States alone, and this value is expected to increase each year as the incidence of KSD rises (7).

In addition to the direct medical burden associated with KSD, patients with KSD are more likely to develop cardiovascular disease (CVD), chronic kidney disease (CKD), and renal insufficiency (8–10). Furthermore, patients with KSD also have an increased burden of comorbid conditions, including dyslipidemia, hypertension, diabetes, and metabolic syndrome (11–14). Both ailments are chronic disorders distinguished by their gradual progression over an extended duration. The production of reactive oxygen species (ROS) and the emergence of oxidative stress (OS) might signify a common route (15). Moreover, additional elements such as elevated sodium consumption, dietary intake of animal-derived proteins, and irregularities in purine metabolism (excessive dietary protein, overexpression of pivotal enzymes) could exert a significant impact (14). Although KSD is not typically considered a life-threatening disease, it can still lead to death, and the number of deaths is increasing with the rising incidence rate in England (5). Complications associated with stones, particularly urosepsis, are the primary factor contributing to mortality (5, 16). Given the association with CVD, the higher risk of death in KSD patients may be primarily due to CVD mortality (8, 16, 17). Therefore, in addition to adequate preoperative preparation for KSD to avoid death related to stone complications, it is crucial to identify controllable risk factors that contribute to or accelerate disease-related mortality.

The main active state of vitamin D in our body is 25-hydroxyvitamin D, which primarily regulates calcium and phosphorus metabolism (18). It is generally known that 25(OH)D facilitates calcium absorption and digestion, which, in turn, increases urinary calcium content and theoretically enables increased kidney stone formation. Despite previous research indicating that elevated concentrations of 25(OH)D and vitamin D supplementation did not increase the risk of KSD (19, 20), recent high-quality studies have demonstrated that both can actually increase the risk of developing KSD (21–23). On the other hand, a deficiency in serum 25(OH)D may increase the risk of developing various diseases, such as CVD, hypertension, obesity, diabetes, and chronic kidney disease (24–26). Higher concentrations of serum 25(OH)D have been proven to be associated with reduced all-cause and cardiovascular disease mortality in various populations, including those with diabetes, osteoporosis, the elderly, and cardiovascular disease (27–31). A review of relevant studies found that vitamin D supplementation only statistically decreased the risk of cancer-related deaths. However, it did not reduce overall mortality or improve cardiovascular disease outcomes (32).

Although serum 25(OH)D has shown potential benefits in reducing mortality risk, there is a lack of research on its effects in the KSD population. To address this gap, our study aimed to evaluate the impact of serum 25(OH)D concentration on mortality in a prospective cohort of KSD patients from the National Health and Nutrition Examination Survey (NHANES) database. Through this investigation, we aim to provide insight into the potential benefits of serum 25(OH)D in reducing mortality risk among KSD patients.

## Methods

### Study population

The NHANES project aims to assess the health and nutritional status of the American population through a combination of physical tests and interviews. In our study, we utilized data from this program to investigate the potential impact of serum 25(OH)D on all-cause mortality in adults with KSD.

**Figure 1:**
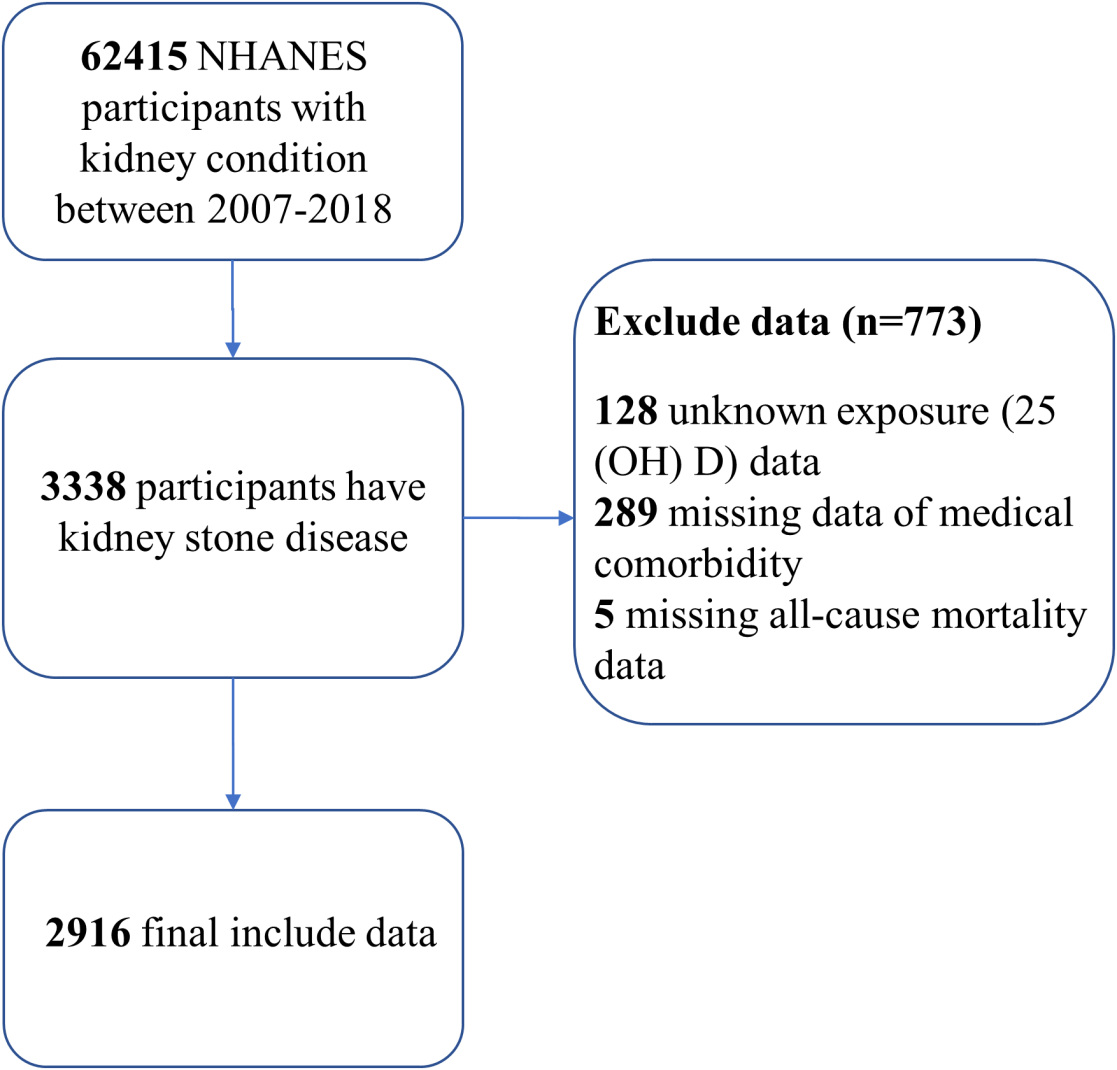
Flow chart of the study.

Our research utilized data from six NHANES cycles spanning from 2007 to 2018. We included individuals aged 20 years or older who had answered a questionnaire regarding their history of kidney stones (“Have you ever had kidney stones?”) (33). Using a standardized liquid chromatography–tandem mass spectrometry (LC-MS/MS) technique, we determined the concentrations of serum 25(OH)D. We excluded participants with unknown exposure to 25(OH)D data (n=128), missing medical comorbidity data (n=289), and those without death follow-up data (n=5). As a result, our analytical cohort consisted of 2916 participants with kidney stones (**Figure 1**).

### Ascertainment of mortality

To determine the mortality status of our participants, we utilized specific study identifiers (SEQN) and probability matching to the National Death Index (NDI) as of December 31, 2018. The National Center for Health Statistics provided further details regarding the matching procedure.

In addition to determining mortality status, we also utilized the 10th revision to the international statistical classification of diseases (ICD-10) to identify disease-specific deaths. Our study’s primary findings focused on mortality from all causes and from specific diseases.

### Assessment of covariates

Data were collected in cycles from 2007 to 2018, including information on sociodemographic traits, physical characteristics, health issues, lifestyle traits, and lab results. Sociodemographic traits included age (less than 60 years and 60 years or older), gender, race (non-Hispanic white, non-Hispanic black, Hispanic, and other), marital status (married or with partner, single), educational attainment (less than high school, high school, and greater than high school), and family poverty ratio (less than 1.3, 1.3 to less than 3.5, and 3.5 or greater). Leisure-time physical activity, defined as engaging in moderate-to-strenuous recreational physical activity during a typical week, was a key lifestyle attribute (2). BMI was selected as the body measurement parameter and was categorized according to clinical guidelines for overweight and obesity: less than 25.0 kg/m2, 25.0-29.9 kg/m2, and 30 kg/m2 or greater. Smoking status was classified as current smoker or non-smoker. Drinking status was determined by whether the individual consumed 12 or more alcoholic beverages per year. A self-reported medical history of cancer, diabetes, hypertension, cardiovascular disease, and stroke was also collected.

The 2007-2018 NHANES also included laboratory blood tests, which were conducted using strict procedures for blood collection and analysis. This study focused on several cardiometabolic markers, including plasma glucose, triglycerides (TG), uric acid, cholesterol, low-density lipoprotein cholesterol (LDL), high-density lipoprotein cholesterol (HDL), C-reactive protein (CRP), glycohemoglobin (HbA1c), and insulin, which were measured at baseline.

### Statistical analysis

To ensure nationally representative estimates, we followed the NHANES analytical reporting criteria and utilized survey analysis methodologies (34). Referring to the Endocrine Society Clinical Practice guidelines, the concentration of serum 25(OH)D was categorized into four groups (<25.0 nmol/L, 25.0–49.9 nmol/L, 50.0–74.9 nmol/L, and ≥75.0 nmol/L) (35). All statistical analyses were performed using STATA version 16.0 and Empower Stats software. To address missing data, we employed the chained equation technique and repeated imputation based on five replicates. Categorical and dichotomous data were presented as percentages, while continuous variables were reported as mean and standard deviation (SD). In order to assess the disparities in clinical characteristics across various groups, we employed chi-square tests for categorical variables and utilized a weighted linear regression model for continuous variables. Person-years were calculated from baseline until the date of death, loss to follow-up, or December 31, 2018, whichever occurred first. We used multivariate Cox regression models to calculate hazard ratios (HR) and 95% confidence intervals (CIs). We constructed three models: (1) non-adjusted; (2) adjusted for age, gender, race, BMI, educational attainment, leisure-time physical activity, marital, family poverty ratio, smoking, and alcohol; and (3) adjusted for age, gender, race, BMI, educational attainment, leisure-time physical activity, marital, family poverty ratio, smoking, alcohol, diabetes, hypertension, stroke, and cardiovascular disease. We conducted additional subgroup analyses to investigate the association between serum 25(OH)D and all-cause mortality and tested for P-values for interactions within each subgroup. Weighted generalized additive model regression and smoothed curve fitting were used to explore the relationship between serum 25(OH)D and all-cause mortality in greater detail. If a non-linear correlation was identified, a two-piecewise linear regression would be used to determine the effect threshold.

## Results

### Baseline characteristics of participants

Among the 2916 adults with KSD, the median age was 58.0 years (interquartile range 26.0 years), and 44.4% were women. Among this group, 26.3% had a deficiency of 25(OH)D (<50 nmol/L), and 64.1% had a deficiency of 25(OH)D (<75 nmol/L). **Table 1** displays the weighted sociodemographic characteristics and some disease conditions of the four groups. Participants with better serum 25(OH)D status had an older age, a higher percentage of non-Hispanic whites, higher diploma and household earnings, and were less likely to have diabetes or be overweight (P<0.01). When comparing cardiometabolic biomarkers, participants with better serum 25(OH)D status were likely to have worse glucose, glycohemoglobin, C-reactive protein (CRP), and insulin and higher HDL-cholesterol (P < 0.01, **Table 2**).

**Table 1:** Baseline characteristics of KSD participants in NHANES data from 2007 to 2018, weighted.

	Serum 25(OH)D concentrations (nmol/L)				
	<25	25-49.9	50-74.9	≥75	P value
Number of participants (%)	99 (3.4)	668 (22.9)	1101 (37.8)	1048 (36.0)	
Age (years)	51.8 ± 15.8	51.5 ± 15.1	50.8 ± 15.1	56.8 ± 15.7	<0.01
Gender (%)					<0.01
Man	36.6	51.6	63.2	48.2	
Woman	63.4	48.4	36.8	51.8	
Race (%)					<0.01
Non-Hispanic white	46.1	60.1	74.9	87.9	
Non-Hispanic black	27.4	14.4	4.0	2.5	
Hispanic	7.5	7.2	7.0	2.3	
Other	19.0	18.2	14.1	7.3	
Body Mass Index (kg/m^2^)					<0.01
<25	6.8	15.6	16.8	24.0	
25 - <30	30.4	27.8	31.9	35.5	
≥30	62.8	56.6	51.3	40.5	
Educational attainment (%)					<0.01
<High school	19.7	17.8	17.8	12.6	
High school	28.2	23.8	22.9	22.5	
>High school	52.1	58.4	59.3	64.9	
Family poverty ratio (%)					<0.01
<1.3	24.4	26.7	22.1	14.6	
1.3 - <3.5	49.4	36.0	36.4	38.4	
≥3.5	26.2	37.3	41.5	47.0	
Leisure-time physical activity (%)					0.02
Inactive	94.9	86.5	16.3	15.4	
Active	5.1	13.5	83.7	84.6	
Marital status (%)					<0.01
Married or with partner	55.0	64.7	70.5	69.4	
Single	45.0	35.3	29.5	30.6	
Alcoholic (%)					0.12
Yes	59.5	69.7	71.4	72.0	
No	40.5	30.3	28.7	28.0	
Smoke (%)					0.61
Yes	46.5	48.5	51.2	52.1	
No	53.5	51.5	48.8	47.8	
Diabetes (%)	27.3	22.5	17.1	17.7	0.01
Hypertension (%)	46.6	47.3	44.3	49.8	0.07
Cardiovascular disease (%)	7.1	7.9	5.1	7.2	0.11
Stroke (%)	5.7	4.3	4.5	4.7	0.95

**Other:** other than non-Hispanic white, non-Hispanic black, and Hispanic, including multiracial.

**Leisure-time physical activity:** moderate or vigorous recreational physical activity.

**Table 2:** Baseline levels of cardiometabolic markers according to serum 25(OH)D concentrations among participants with kidney stones.

	Serum 25(OH)D concentrations (nmol/L)				P value
	<25.0	25.0-49.9	50.0-74.9	≥75	
Glucose, mg/dL	109 ± 41	115 ± 51	106 ± 37	104 ± 37	<0.01
Triglycerides, mg/dL	151 ± 91	160 ± 112	183 ± 165	170 ± 197	0.04
Uric acid, mg/dL	5.6 ± 1.7	5.6 ± 1.4	5.6 ± 1.4	5.6 ± 1.5	0.99
Cholesterol, mg/dL	196 ± 59	190 ± 42	191 ± 40	194 ± 44	0.24
LDL-cholesterol (mg/dL)	104 ± 34	116 ± 35	114 ± 33	112 ± 36	0.04
HDL-cholesterol, mg/dL	50.5 ± 14.0	48.6 ± 13.8	47.2 ± 13.5	53.1 ± 16.3	<0.01
CRP, mg/L	7.2 ± 12.9	5.3 ± 8.5	5.3 ± 10.8	4.1 ± 8.6	<0.01
Glycohemoglobin, %	6.1 ± 1.3	6.1 ± 1.4	5.8 ± 1.1	5.8 ± 0.9	<0.01
Insulin, ρmol/L	126 ± 148	110 ± 106	114 ± 100	100 ± 103	<0.01

Mean ± SD for continuous variables: P-value was calculated by weighted linear regression model.

### Relationships of 25(OH)D concentration with mortality

During the average follow-up of 6.4 years (range, 0.17-13.2 years), encompassing a total of 18,859 person-years, there were 375 all-cause fatalities. We conducted three Cox regression models, as presented in **Table 3**, to investigate the independent impact of serum 25(OH)D status on mortality. No relationship between serum vitamin D concentration and mortality was observed before adjustment. In adjusted model, we discovered a negative association between serum 25(OH)D concentrations and all-cause mortality. The hazard ratios (HRs) and 95% confidence intervals (CIs) of Model 3 for serum 25(OH)D severe deficiency to sufficient (25.00, 25.00-49.99, 50.00-74.99, and 75.00 nmol/L) were, respectively, 1.00 (reference), 0.62 (0.34, 1.13), 0.63 (0.35, 1.13), and 0.48 (0.26,0.87) and P for trend was 0.02. For CVD or cancer mortality (P trend = 0.68), the HR and 95% CI were not statistically significant, and P for trend was 0.68 and 0.08 respectively. Adjustments for LDL, HDL, TG, and CRP did not significantly alter the results (**Table 4**).

**Table 3:** HR (95% CIs) for all-cause and cause-specific mortality according to serum 25(OH)D concentrations among participants with kidney stones.

	Serum 25(OH)D concentrations (nmol/L)				P for trend
	<25	25-49.9	50-74.9	≥75	
All-cause mortality					
Number of deaths (%) Model 1 HR (95% CI) P Model 2 HR (95% CI) P Model 3 HR (95% CI) P	19 (19.2) Reference Reference Reference	79 (11.8) 0.58 (0.35, 0.95) 0.03 0.56 (0.32, 0.99) 0.04 0.62 (0.34, 1.13) 0.12	142 (12.9) 0.65 (0.40, 1.05) 0.08 0.60 (0.34, 1.03) 0.06 0.63 (0.35, 1.13) 0.12	135 (12.9) 0.69 (0.43, 1.12) 0.13 0.50 (0.29, 0.87) <0.01 0.48 (0.26, 0.87) 0.02	0.68 0.04 0.02
CVD mortality					
Number of deaths (%) Model 1 HR (95% CI) P Model 2 HR (95% CI) P Model 3 HR (95% CI) P	7 (7.1) Reference Reference Reference	20 (3.0) 0.96 (0.40, 2.31) 0.92 0.84 (0.28, 2.52) 0.76 0.55 (0.16, 1.95) 0.36	32 (2.9) 0.59 (0.25, 1.38) 0.23 0.52 (0.17, 1.61) 0.25 0.21 (0.05, 0.85) 0.03	24 (2.3) 1.21 (0.51, 2.85) 0.67 1.29 (0.40, 4.21) 0.67 0.91 (0.23, 3.63) 0.89	0.58 0.27 0.68
Cancer mortality					
Number of deaths (%) Model 1 HR (95% CI) P Model 2 HR (95% CI) P Model 3 HR (95% CI) P	7 (7.1) Reference Reference Reference	13 (2.0) 0.76 (0.21, 2.70) 0.67 0.68 (0.12, 3.72) 0.65 0.67 (0.12, 3.78) 0.65	20 (1.8) 0.45 (0.13, 1.58) 0.21 0.23 (0.05, 1.02) 0.05 0.33 (0.07, 3.78) 0.64	39 (3.7) 0.45 (0.13, 1.52) 0.20 0.20 (0.05, 0.82) 0.03 0.32 (0.07, 1.42) 0.13	0.08 0.02 0.08

**Model 1:** Non-adjusted model.

**Model 2:** Adjusted for age, gender, race, BMI, educational attainment, leisure-time physical activity, marital status, family poverty ratio, smoking status, and alcohol status.

**Model 3:** Adjusted for age, gender, race, BMI, educational attainment, leisure-time physical activity, marital status, family poverty ratio, smoking status, alcohol status, diabetes, hypertension, stroke chronic kidney disease, cancer, and cardiovascular disease.

**Table 4:** Hazard ratios (95% CIs) of all-cause mortality according to serum 25(OH)D concentrations among KSD with further adjustment of blood lipids and CRP.

	Serum 25(OH)D concentrations (nmol/L)				P for trend
	<25	25-49.9	50-74.9	≥75	
All-cause mortality					
Number of deaths (%) Model 1 HR (95% CI) P Model 2 HR (95% CI) P Model 3 + blood lipids HR (95% CI) P	19 (19.2) Reference Reference Reference	79 (11.8) 0.58 (0.35, 0.95) 0.03 0.56 (0.32, 0.99) 0.04 0.61 (0.33, 1.11) 0.10	142 (12.9) 0.65 (0.40, 1.05) 0.08 0.60 (0.34, 1.03) 0.06 0.61 (0.33, 1.09) 0.09	135 (12.9) 0.69 (0.43, 1.12) 0.13 0.50 (0.29, 0.87) <0.01 0.44 (0.24, 0.81) <0.01	0.68 0.04 <0.01
Model 3 + CRP HR (95% CI) P	Reference	0.62 (0.34, 1.12) 0.11	0.62 (0.35, 1.12) 0.12	0.48 (0.26, 0.87) 0.02	0.02
Model 3 + blood lipids + CRP HR (95% CI) P	Reference	0.60 (0.33, 1.10) 0.10	0.60 (0.33, 1.10) 0.09	0.44 (0.25, 0.81) <0.01	<0.01

**Model 1:** Non-adjusted model.

**Model 2:** Adjusted for age, gender, race, BMI, educational attainment, leisure-time physical activity, marital status, family poverty ratio, smoking status, and alcohol status.

**Model 3:** Adjusted for age, gender, race, BMI, educational attainment, leisure-time physical activity, marital status, family poverty ratio, smoking status, alcohol status, diabetes, hypertension, stroke chronic kidney disease, cancer, and cardiovascular disease.

### The detection of non-linear relationships

**Figure 2:**
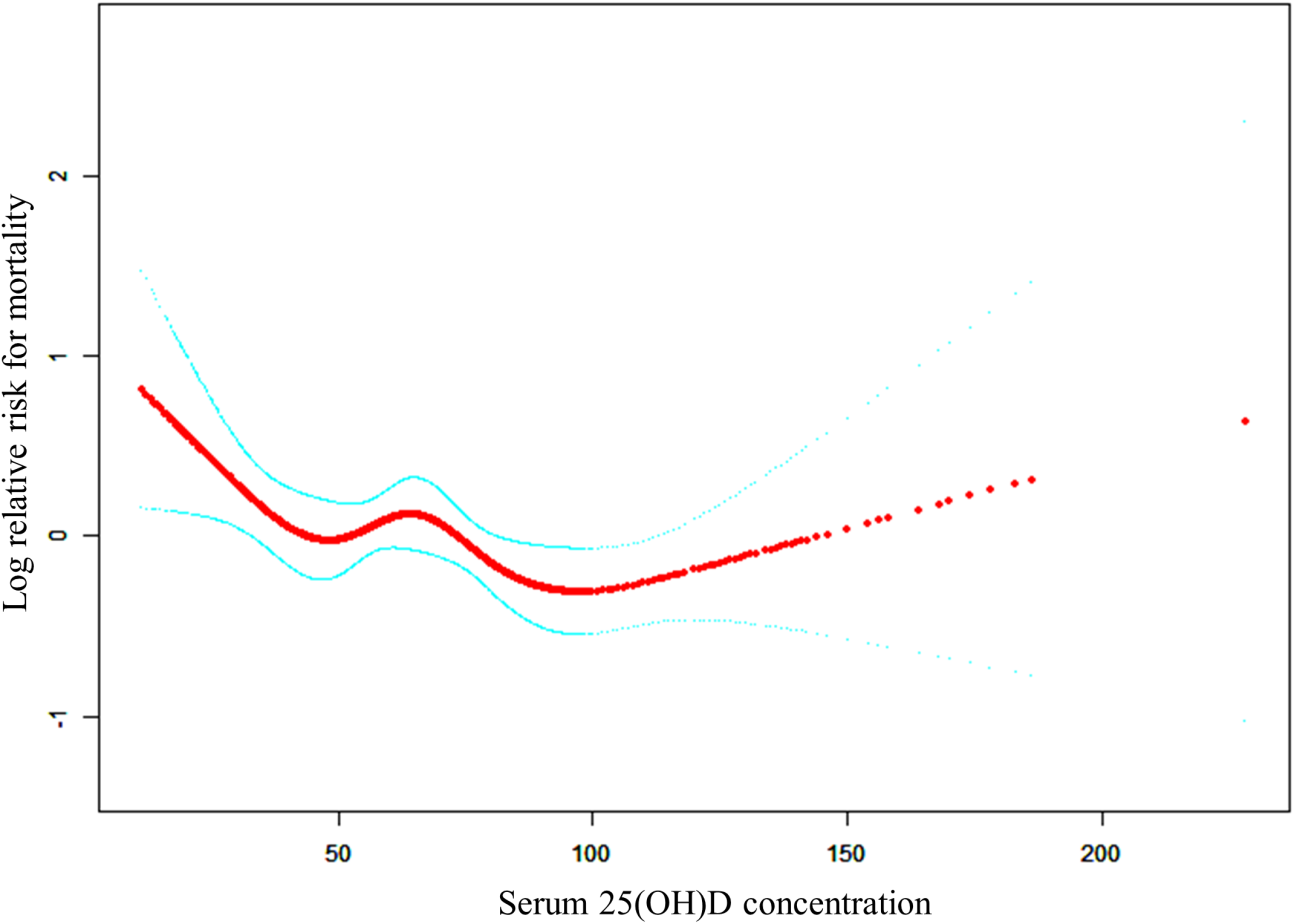
The non-linear relationship between serum 25(OH)D concentrations and the risk of all-cause mortality.

The adjusted smooth curve model (Model 3) revealed a non-linear association (**Figure 2**). To further investigate the nonlinear relationship between serum 25(OH)D concentrations and all-cause and CVD mortality in patients with KSD, the threshold effect analysis revealed the inflection points for all-cause mortality were 27.7 nmol/L. For serum 25(OH)D concentrations below 27.70 nmol/L, each unit increase was associated with a 7% reduction in the risk of all-cause mortality (HR 0.93; 95% CI: 0.88, 0.99) (**Table 5**). When serum 25(OH)D concentrations exceeded 27.70 nmol/L, there was no association with all-cause mortality (HR 0.99; 95% CI: 0.99, 1.00).

**Table 5:** Threshold effect analysis of serum 25(OH)D concentrations on all-cause and cancer mortality in kidney stone patients.

Population	Cut-off value	Adjusted HR (95% CI), P	Log likelihood ratio
All-cause mortality	25(OH)D concentrations < 27.7 nmol/L	0.93 (0.88, 0.99), 0.02	0.05
	25(OH)D concentrations≥27.7 nmol/L	0.99 (0.99, 1.00), 0.15	
Age≥60 years	25(OH)D concentrations < 123 nmol/L	0.99 (0.98, 0.99), <0.01	0.01
	25(OH)D concentrations≥123 nmol/L	1.03 (1.00, 1.05), 0.02	
Non-Hispanic white	25(OH)D concentrations < 96 nmol/L	0.99 (0.98, 0.99), 0.02	0.01
	25(OH)D concentrations≥96 nmol/L	1.01 (0.99, 1.02), 0.07	
With Cancer	25(OH)D concentrations < 31.1nmol/L	0.89 (0.82, 0.98), 0.01	0.03
	25(OH)D concentrations≥31.1 nmol/L	0.99 (0.99, 1.00), 0.89	
With CVD	25(OH)D concentrations < 48.5nmol/L	0.94 (0.90, 0.97), <0.01	<0.01
	25(OH)D concentrations≥48.5 nmol/L	1.02 (1.00, 1.13), 0.01	

Adjusted for age, gender, race, BMI, educational attainment, leisure-time physical activity, marital status, family poverty ratio, smoking status, alcohol consumption, diabetes, hypertension, stroke, chronic kidney diseases, cancer, and cardiovascular disease.

### Stratified analyses

**Figure 3:**
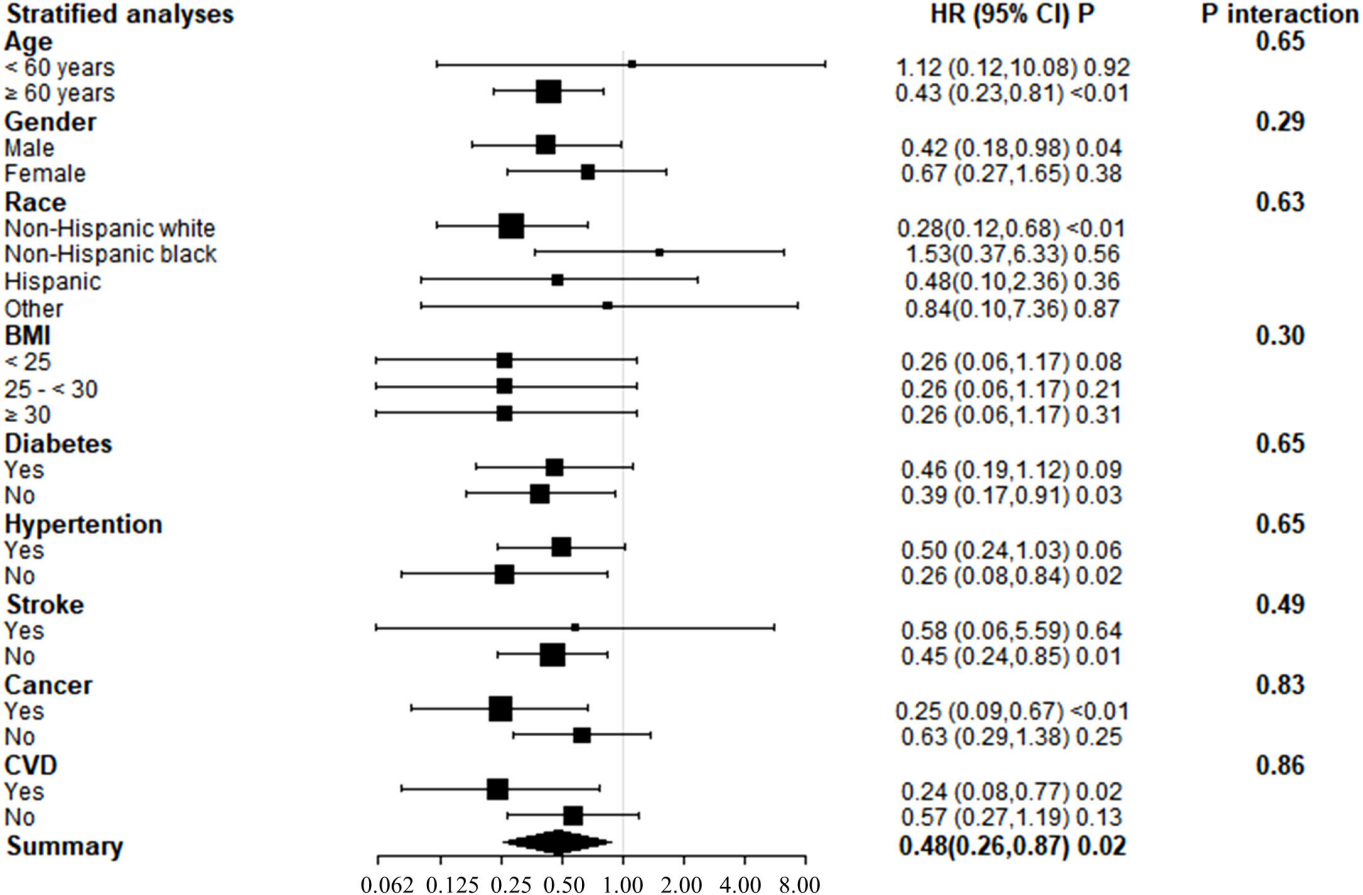
Forest plots of stratified analyses of 25(OH)D and all-cause mortality.

Subgroup analyses of adjusted model 3 were conducted to determine the benefit of increased serum 25(OH)D concentrations on KSD patient survival, stratified by age, gender, race, BMI, diabetes, hypertension, stroke, cancer, and CVD (**Figure 3**). Serum 25(OH)D concentrations and stratification factors did not show significant interaction. Although the interaction was not clear, our findings revealed stronger inverse associations between 25(OH)D concentrations and all-cause mortality in older adults (>60 years), non-Hispanic whites, and patients with cancer or cardiovascular disease. The threshold effect analysis found the inflection points were 123 nmol/L, 31.0nmol/L, 31.1nmol/L, and 48.5nmol/L in participants ≥ 60 years old, who were Non-Hispanic white, with cancer, and with CVD, respectively (**Table 5**). For individuals over the age of 60 and Non-Hispanic white individuals, a decrease of 1 unit in serum 25(OH)D concentrations, when below 123.0 nmol/L or 96.0 nmol/L, respectively, was associated with a 1% decrease in the risk of all-cause mortality. The hazard ratios were 0.98 (95% CI 0.98, 0.99) and 0.99 (95% CI 0.98, 0.99) for the two groups, respectively.

### Sensitivity analysis

The results remained robust in sensitivity analyses that excluded cases with less than 1 or 2 years of follow-up (**Supplementary Table S1**). The fully adjusted hazard ratio (HR) of all-cause mortality rate for serum 25(OH)D concentrations ≥75 nmol/L relative to <25nmol/L, excluding deaths at 1 year, was 0.56 (95% CI: 0.33, 0.97), and excluding deaths at 2 years, it was 0.52 (95% CI: 0.30, 0.92), compared to 0.48 (95% CI: 0.26, 0.87) for all deaths. In a calculation that took into account the number of deaths at > 3 y, the estimated HR for the all-cause mortality rate was 0.57 (95% CI: 0.31, 1.04, P=0.07).

## Discussion

In this study, we were able to determine the impact of 25(OH)D concentrations on all-cause mortality in the KSD population using follow-up data from the NHANES death cohort. We found that higher concentrations of serum 25(OH)D were strongly associated with lower all-cause mortality within a certain range, independent of traditional risk factors such as lifestyle, BMI, cardiometabolic indicators, diabetes, hypertension, stroke, CVD, CKD, and cancer. This conclusion was further supported by other stratified analyses. In addition, KSD patients with higher 25(OH)D concentrations had lower levels of inflammatory biomarkers and better lipid profiles. Even after accounting for these factors, 25(OH)D still had an impact on all-cause mortality. Our findings suggest potential therapeutic interventions and may serve as dietary guidelines and clinical intervention references for reducing all-cause mortality in KSD patients.

Low serum 25(OH)D concentrations, especially 25(OH)D deficiency, have been associated with numerous diseases and all-cause mortality (18, 24–28, 36). Recently, a large study provided real-world evidence to support the effectiveness of vitamin D supplementation in reducing both overall mortality and mortality related to specific causes (37). As a result, there has been a significant increase in the prescription of vitamin D, even for healthy individuals. However, a recent meta-analysis of randomized clinical trials and a Cochrane review have shown that vitamin D and calcium supplementation did not have the benefit of reducing all-cause mortality but reduced the risk of cancer death (32, 38, 39). Moreover, the RCT found a significant reduction in cancer mortality after omission of the first one and two years of data (40). It indicated that the long-term effects of vitamin D supplementation play a more significant role in reducing the risk of death from cancer. Additionally, epidemiological studies have found that taking vitamin D supplements increases the risk of KSD in predisposed individuals (22, 23, 38). Our study indicated that raising vitamin D concentrations alone may not be sufficient to prevent CVD or cancer death in KSD patients, although it was associated with all-cause mortality. Other factors may play a more significant role in the development of CVD or cancer death in this population. The conflicting results may be due to differences in study populations, interventions, outcome definitions, and analytical methods. Therefore, it is crucial to consider these differences when drawing conclusions about the appropriate concentrations of vitamin D for overall health and longevity to avoid inappropriate comparisons. Our study suggests that there is an optimal range of vitamin D concentrations for health, and both low and high concentrations may increase the risk of mortality. Once the serum vitamin D concentration reaches a specific threshold (27.7nmol/L in our study) through supplements, the mortality rate will not be further decreased. Previous studies have been conducted on individuals who are either at high risk for cardiovascular disease or the general population, but there have been no investigations on the role of vitamin D in mortality among individuals with KSD who are more prone to developing chronic kidney disease (25, 41).

There is disagreement regarding the role of 25(OH)D in KSD. Previous studies from the Third NHANES have shown that high concentrations of 25(OH)D were not associated with KSD, and kidney stone prevalence did not increase the risk of all-cause and CVD death (19, 42). However, a meta-analysis reported that there was a significant increase in circulating 25(OH)D concentrations only in hypercalciuria stone formers (65.7 nmol/L vs 54.6 nmol/L for hypercalciuria stone formers and controls, respectively), while there was no significant elevation in 25(OH)D concentrations of total stone formers (72.8 nmol/L vs 79.3 nmol/L for total stone formers and controls, respectively) (23). The evidence supporting the role of serum 25(OH)D and vitamin D supplementation in promoting kidney stone formation primarily comes from a Mendelian study, which has the specific ability to establish causal relationships. This study indicates that the impact of vitamin D supplements was limited to the duration of the experimental period, while higher concentrations of 25(OH)D circulating throughout one’s lifetime have a more significant influence on the occurrence of kidney stones (21). Long-term supplementation with vitamin D alone may increase the risk of KSD by increasing 25(OH)D concentrations. The conflicting results among studies regarding the relationship between vitamin D and kidney stone formation may be attributed to the interference of major risk factors associated with kidney stone formation. For example, other nutritional factors, including dietary protein, carbohydrates, oxalates, calcium, and sodium chloride, can also regulate the risk of urinary conditions and increase the risk of kidney stone formation (43). Although there is controversy regarding the conclusion that supplementing vitamin D alone increases the risk of hypercalciuria and kidney stones (44, 45), it is clear that there is an increased risk of kidney stone formation when vitamin D is supplemented along with calcium (22, 46). Additionally, strong evidence has shown that low urine output and dehydration are the common risks of all stone types (6, 47). This is because adequate fluid intake helps to dilute the abnormal urine components that contribute to stone formation, such as hypercalciuria, hyperoxaluria, and other factors. It is our belief that individuals with a genetic susceptibility to high serum vitamin D concentrations are more prone to developing hypercalciuria, thereby increasing the risk of kidney stones. A recent meta-analysis has also found that supplemental vitamin D doses of 3200-4000 IU/d may lead to an increased risk of hypercalcemia and other adverse events, but only in a small subset of individuals (48). Similarly, the higher risk of dying from any cause, including CVD, in KSD patients may be related to unique population characteristics and related comorbidities such as diabetes and CVD (42). To better understand how vitamin D affects all-cause and specific mortality in the KSD population, we conducted this study and found that 25(OH)D insufficiency (<27.7nmol/L) was associated with an increased risk of all-cause mortality in the KSD population after adjusting for unique demographics and associated comorbidities. However, this association was not present for CVD and cancer-specific causes of death.

There are various factors that could account for the decrease in all-cause mortality risk, particularly cardiovascular disease mortality, associated with a reduction in 25(OH)D concentrations. As previously mentioned, low serum concentrations of 25(OH)D have been linked to numerous diseases that increase the risk of premature death, including hypertension, dyslipidemia, diabetes, chronic kidney disease, and, notably, cardiovascular disease (18, 25, 26, 49–51). Hence, the findings from the two additional studies conducted on individuals with diabetes or osteoarthritis indicate that a deficiency in 25(OH)D primarily elevates the risk of mortality from cardiovascular disease (27, 28). On other hand, meat is an important source of vitamin D. Vegetarians and vegans have been shown to have lower plasma concentrations of 25(OH)D compared to individuals who consume meat and fish (52). Additionally, red meat and processed meat increase the risk of chronic diseases such as diabetes, chronic kidney disease, and coronary heart disease (53–55). Hence, we should also take into consideration whether the population that shows an increase in serum vitamin D concentrations due to meat consumption may obscure the health effects of vitamin D. The mechanism by which 25(OH)D reduces the risk of mortality from cardiovascular disease may involve the inhibition of renin synthesis, reduction of angiotensin II production, improvement of pancreatic beta-cell function, endothelial protective effects, and immunomodulatory activities, as suggested by previous research (18). Furthermore, 25(OH)D may improve adverse cholesterol metabolism, reduce inflammatory parameters, and enhance myocardial function and glucose metabolism (56, 57).

Interestingly, it was observed that in the KSD population with 25(OH)D insufficiency, there was no statistically significant association between 25(OH)D and mortality from cardiovascular disease. Our study revealed that individuals with KSD who had lower concentrations of 25(OH)D also had higher levels of CRP, indicating that the impact of 25(OH)D on mortality may be partially mediated through inflammatory pathways. Moreover, there is mounting evidence that 25(OH)D deficiency may heighten the risk of fatal events resulting from infections (58–61). A recent systematic review on mortality in KSD patients revealed that 21% of the reported mortality cases out of 2550 were related to surgical procedures, with sepsis being one of the primary causes of mortality (16). In fact, more than half of the deaths in patients who underwent intervention for KSD were attributed to sepsis (62). It is worth noting that other causes of death, such as cardiac-related, respiratory-related, and multiorgan failure, could also be attributed to sepsis. Based on this finding, it is possible that the higher mortality rate in KSD patients may be due to the fact that infections resulting from stone obstruction or intervention are more likely to progress to sepsis. Vitamin D, which can have activating or inhibiting effects on immune responses in the body, has been linked to susceptibility to infections. Most studies have demonstrated a strong association between sepsis and vitamin D deficiency, with sepsis patients who have low concentrations of vitamin D being more prone to developing secondary infections (63–66). However, rather than being a direct cause of mortality, vitamin D deficiency may be an indicator of other unfavorable health outcomes that could lead to premature death. Vitamin D also exerts an influence on other diseases related to the urinary system. Numerous studies have demonstrated the positive impact of vitamin D on reducing prostate volume and PSA levels and improving symptoms associated with benign prostatic hyperplasia (BPH) (67–69). In addition, vitamin D deficiency increases the risk of overactive bladder and urinary incontinence, and vitamin D supplementation reduces the risk of urinary incontinence (70). This can also explain why studies have observed lower concentrations of vitamin D in men with lower urinary tract symptoms (LUTS) (71). The impact of vitamin D is well described in certain tumors; however, the findings regarding the role of vitamin D in prostate cancer and serum PSA levels are contradictory and lack consistency (72, 73). Moreover, there has a possible relationship between vitamin D and urinary tract infection (UTI), erectile dysfunction (ED), and the male reproductive system (74–78). Therefore, further research is needed to elucidate how 25(OH)D impacts mortality in KSD patients.

Our study is the first to investigate the correlation between serum 25(OH)D concentrations and all-cause mortality in a large, nationally representative sample of KSD patients, and we were able to control for a range of potential confounding variables using comprehensive data from the NHANES survey. However, there are several limitations to our study. Firstly, we relied on self-reported and recalled disease data, which may have introduced memory bias. Secondly, as NHANES is a cross-sectional survey, we were unable to track changes in confounding variables over time, especially the serum 25(OH)D concentration. **Supplementary Table 1** also confirms that the strength of our study results weakens or even loses statistical significance with increasing follow-up time. Finally, we were unable to determine the impact of kidney stone location, size, and composition on all-cause mortality. Further research that includes repeated follow-up and additional variables is needed to better understand the relationship between variations in 25(OH)D concentrations and mortality risk in KSD patients.

## Conclusion

Our study found that after adjusting for multiple variables, serum 25(OH)D insufficiency (less than 27.7nmol/L) was associated with an increased risk of all-cause mortality in KSD patients, but this association was not observed for specific causes of death such as CVD and cancer. This effect may be due in part to the inflammatory pathways of vitamin D. Our results suggest that monitoring and preventing serum 25(OH)D deficiency may have potential benefits in reducing premature death in KSD patients.

## Data availability statement

Publicly available datasets were analyzed in this study. This data can be found here: https://www.cdc.gov/nchs/nhanes/index.htm.

## Ethics statement

Approval of the study from the National Center of Health and Statistics Research ethics review board was waived because the research relied on publicly used, de-identified secondary data.

## Author contributions

HC and ZZ made contributions to the conception and design of the work; MG analyzed and interpreted the data and drafted the work; ML and JC substantively revised the work. All authors have approved the submitted version and agreed both to be personally accountable for their own contributions and to ensure that questions related to the accuracy or integrity of any part of the work, even ones in which the author was not personally involved, are appropriately investigated, resolved, and the resolution documented in the literature.
