# Supplementary material for: Association of serum 25-hydroxyvitamin D concentrations with all-cause mortality among individuals with kidney stone disease: the NHANES database prospective cohort study
Source: Front Endocrinol (Lausanne). 2023 Oct 3;14:1207943. doi: 10.3389/fendo.2023.1207943 (PMC10579890; doi:10.3389/fendo.2023.1207943)
Supplement: Supplementary file 1 [file Table_1.docx]

**Supplementary Table 1 HR (95% CIs) for all-cause according to serum 25(OH)D concentrations among participants with kidney stones.**

|  | **Serum 25(OH)D concentrations (nmol/L)** | | | |
| --- | --- | --- | --- | --- |
|  | **<25** | **25-49.9** | **50-74.9** | **≥75** |
| **Follow-up > 1 year** |  |  |  |  |
| Number of deaths (%) Model 1  HR (95% CI) P  Model 2  HR (95% CI) P  Model 3  HR (95% CI) P | 17 (17.5)  Reference  Reference  Reference | 67 (10.2)  0.54 (0.32, 0.93) 0.03  0.57 (0.33, 0.99) 0.048  0.66 (0.39, 1.13) 0.13 | 131 (12.0)  0.66 (0.40, 1.11) 0.12  0.62 (0.36, 1.07) 0.09  0.75 (0.44, 1.27) 0.28 | 121 (11.7)  0.70 (0.42, 1.16) 0.17  0.48 (0.28, 0.84) <0.01  0.56 (0.33, 0.97) 0.037 |
| **Follow-up > 2 year** |  |  |  |  |
| Number of deaths (%) Model 1  HR (95% CI) P  Model 2  HR (95% CI) P  Model 3  HR (95% CI) P | 15 (17.2)  Reference  Reference  Reference | 57 (9.5)  0.52 (0.29, 0.93) 0.03  0.54 (0.30, 0.97) 0.04  0.63 (0.35, 1.11) 0.11 | 120 (12.1)  0.69 (0.40, 1.19) 0.18  0.63 (0.36, 1.10) 0.11  0.76 (0.44, 1.31) 0.32 | 100 (10.9)  0.66 (0.38, 1.14) 0.13  0.44 (0.25, 0.79) <0.01  0.52 (0.30, 0.92) 0.02 |
| **Follow-up > 3 year** |  |  |  |  |
| Number of deaths (%) Model 1  HR (95% CI) P  Model 2  HR (95% CI) P  Model 3  HR (95% CI) P | 13 (16.7)  Reference  Reference  Reference | 44 (8.0)  0.46 (0.24, 0.86) 0.02  0.48 (0.26, 0.91) 0.02  0.57 (0.31, 1.07) 0.08 | 104 (11.8)  0.67 (0.38, 1.24) 0.21  0.64 (0.35, 1.18) 0.15  0.80 (0.44, 1.46) 0.47 | 88 (11.0)  0.67 (0.37, 1.21) 0.18  0.45 (0.24, 0.85) 0.01  0.57 (0.31, 1.04) 0.07 |

**Model 1:** Non-adjusted model;

**Model 2:** Adjust for: age, gender, race, BMI, educational attainment, leisure-time physical activity, marital status, family poverty ratio, smoking status, alcohol status.

**Model 3:** Adjust for: age, gender, race, BMI, educational attainment, leisure-time physical activity, marital status, family poverty ratio, smoking status, alcohol status, diabetes, hypertension, stroke chronic kidney disease, cancer and cardiovascular disease.
